# Viral and Cellular Requirements for the Nuclear Entry of Retroviral Preintegration Nucleoprotein Complexes

**DOI:** 10.3390/v5102483

**Published:** 2013-10-07

**Authors:** Kenneth A. Matreyek, Alan Engelman

**Affiliations:** Department of Cancer Immunology and AIDS, Dana-Farber Cancer Institute, and Department of Medicine, Harvard Medical School, Boston, Massachusetts, USA; E-Mail: matreyek@fas.harvard.edu

**Keywords:** HIV, lentivirus, nuclear import, nuclear pore complex, capsid, TNPO3, CPSF6, NUP153, NUP358.

## Abstract

Retroviruses integrate their reverse transcribed genomes into host cell chromosomes as an obligate step in virus replication. The nuclear envelope separates the chromosomes from the cell cytoplasm during interphase, and different retroviral groups deal with this physical barrier in different ways. Gammaretroviruses are dependent on the passage of target cells through mitosis, where they are believed to access chromosomes when the nuclear envelope dissolves for cell division. Contrastingly, lentiviruses such as HIV-1 infect non-dividing cells, and are believed to enter the nucleus by passing through the nuclear pore complex. While numerous virally encoded elements have been proposed to be involved in HIV-1 nuclear import, recent evidence has highlighted the importance of HIV-1 capsid. Furthermore, capsid was found to be responsible for the viral requirement of various nuclear transport proteins, including transportin 3 and nucleoporins NUP153 and NUP358, during infection. In this review, we describe our current understanding of retroviral nuclear import, with emphasis on recent developments on the role of the HIV-1 capsid protein.

## 1. Introduction

While some viruses go through the intracellular portions of their life cycle exclusively in the cytoplasm of the host cell, others have adapted to utilize the unique features offered by the nucleus. This includes viruses of the family *Retroviridae*, which differ from other animal viruses by requiring integration into host chromosomes as an obligate step of their life cycles. Aside from the periods of time when animal cells are actively dividing, cell chromosomes and the associated nucleoplasm are separated from the cytoplasm by two sets of adjoining membranes, together referred to as the nuclear envelope. The family *Retroviridae* is comprised of seven viral genera, including α through ε, lenti-, and spuma-. The γ-retrovirus Moloney murine leukemia virus (MLV) and lentivirus HIV-1 are historic model systems for the study of retroviral nuclear import due to their contrasting phenotypes under certain conditions of viral infection. MLV is thought to require the dissolution of the nuclear envelope to gain access to host chromosomes, as infection by MLV is highly dependent on the cycling state of the cells that are being infected [1]. Contrastingly, HIV-1 productively infects post-mitotic cell types [2], and accordingly possesses a mechanism to bypass the intact nuclear envelope to gain access to the sites of integration.

**Figure 1:**
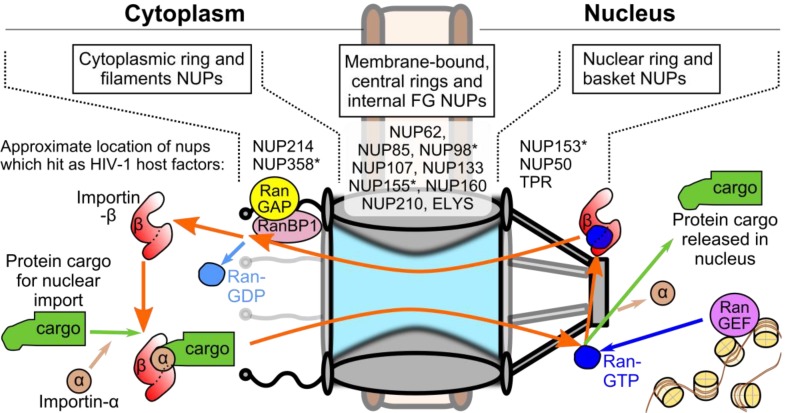
Schematic of the nuclear pore complex (NPC) and classical nuclear import pathway. (top) General NPC substructures and locations of nucleoporins (NUPs) that scored as potential HIV-1 co-factors in genome-wide RNA interference screens [17,18,19,20]. Asterisks denote NUPs that scored in more than one screen. (bottom) The Ran-based nuclear import cycle. Import protein cargo binds to a karyopherin (KPN) β protein, oftentimes bridged by a member of the KPN α protein family (KPN β1, which is also referred to as importin β1, and KPN α2 or importin α1, depicted, are canonical members of each protein family). KPN β1 ferries the complex through the NPC channel. The engagement of KPN β1 by Ran-GTP concentrated at the nuclear basket releases the KPN α-cargo complex into the nucleus. KPN β1 becomes free to bind additional import cargo after Ran dissociates from it upon RanBP1 binding and Ran-GTP hydrolysis, stimulated by RanGAP concentrated at the cytoplasmic filaments.

HIV-1 nucleoprotein complexes are believed to enter the nucleoplasm by passing through nuclear pore complexes (NPCs), which form stable channels through the nuclear envelope during interphase and gate-keep the trafficking of molecules between the cell nucleus and cytoplasm (Figure 1) [3]. Each NPC is composed of ~30 different protein constituents called nucleoporins (NUPs) [4,5,6], which are found in various multiples of eight to yield the 120 MDa tubular structures found in animal cells [7]. Transport through the NPC is highly selective; molecules less than ~9 nm in diameter are able to passively diffuse through the channel, while those up to ~39 nm must be actively transported by interacting with specific carrier proteins [8]. Protein cargos are most often imported by members of the karyopherin (KPN) β superfamily [9]. These proteins pass through the pore by interacting with phenylalanine-glycine (FG) motifs that are found in highly flexible domains present in about one-third of the NUPs and line the inner channel of the NPC structure. Though the precise biophysical mechanism of nuclear transport is not completely understood, it is clear that transport is largely dictated by the properties exerted by these FG-containing domains [10].

Directionality of nuclear translocation is governed by the gradient of the small Ras-related nuclear (Ran) GTPase protein, which is established through the asymmetric distribution of two Ran co-factors on opposite sides of the nuclear envelope (Figure 1). Regulator of chromosome condensation (RCC) 1, which is also referred to as Ran guanine nucleotide exchange factor (RanGEF), achieves nuclear compartmentalization through its association with nucleosomes [11], while Ran GTPase activating protein (RanGAP) is found associated with NUP358 filaments emanating from the cytoplasmic face of the NPC [12]. The classical mechanism of protein nuclear import is governed by two features: Ran binds KPN β proteins when it is complexed with guanosine triphosphate (GTP) rather than guanosine diphosphate (GDP), and protein cargos undergoing nuclear import preferentially bind Ran-free KPN β proteins. Due to the Ran gradient, KPN β proteins bind their import cargos within the cytoplasm, and ferry their cargos through the nuclear pore by interacting with the FG barrier [13]. Once in the nucleus, abundantly present RanGTP binds the KPN β protein, freeing the protein cargo within the nucleus. RanGTP-bound KPN β proteins are able to transit back into the cytoplasm, where Ran binding protein (RanBP) 1 and RanGAP stimulate the conversion of RanGTP to RanGDP [14] and dissociate Ran from the KPN β protein, completing the import cycle. Protein nuclear export cargos differ by preferentially binding RanGTP-bound carrier proteins, such as exportin (XPO) 1, to form ternary complexes that are released into the cytoplasm when Ran is freed upon binding with RanBP1/2 [15,16].

## 2. Measurements of the HIV-1 Nucleoprotein Substrate for Nuclear Import

**Figure 2:**
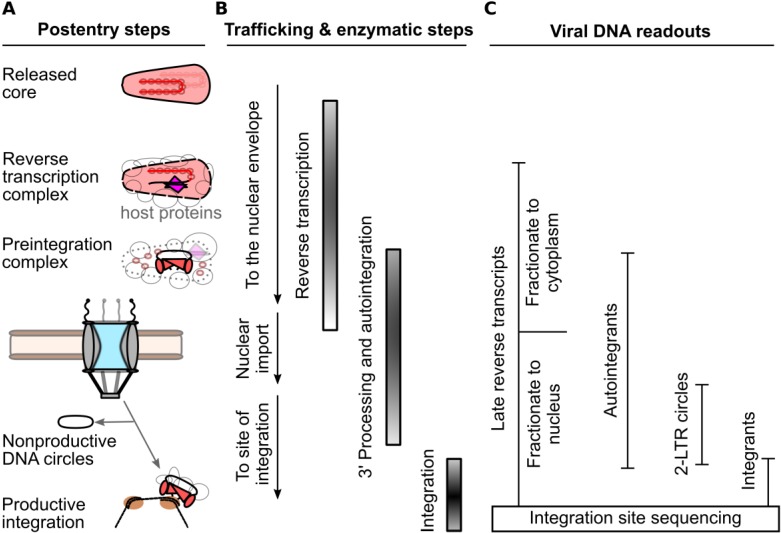
PCR-based methods for detection of post-entry to viral DNA integration steps of HIV-1 infection. **(A)** Generalized replication intermediates and byproducts leading up to integration. **(B)** Order of viral trafficking and RT and IN enzymatic steps. **(C)** Summary of viral DNA species that serve as markers for the various infection intermediates and byproducts.

Mature HIV-1 virions harbor a relatively full complement of viral proteins, including *gag*- (matrix, MA; capsid, CA; nucleocapsid, NC; p6) and *pol*- (protease, PR; reverse transcriptase, RT; and integrase, IN) encoded proteins, as well as a handful of accessory proteins (Vif, Vpr, and Nef). CA is composed of two independently folded protein domains, the N-terminal domain (NTD) and C-terminal domain (CTD), which are separated by a flexible linker [21]. During particle maturation, approximately one-half of the complement of CA protein condenses into a conical shell that is predominantly comprised of hexameric CA rings; twelve pentameric rings afford shape declinations necessary to enclose retroviral CA shells [22,23,24,25]. The biological significance of the remaining CA proteins that are not incorporated into the core is currently unknown. The mature core shell encases the viral components that are necessary to complete the early steps of retroviral infection, which includes the two copies of the viral RNA in complex with NC, RT, and IN. Shortly after viral-cell fusion, the virus reverse transcribes its genome in the context of a subviral complex that is commonly referred to as the reverse transcription complex (RTC) (Figure 2) [26]. DNA synthesis likely triggers CA shell disassembly, as prevention of reverse transcription can delay the steps of core uncoating [27,28]. As the CA core begins to disassemble, some viral proteins diffuse away from the now permeable CA shell [29]. The combination of CA core disassembly and additional host protein recruitment increases the size of the RTC to an estimated ~100–250 nm in diameter [30,31,32]. The number of complete, or near-complete reverse transcribed genomes in a population of infected cells can be readily measured by quantitative PCR, most commonly with a primer pair that generates an amplicon spanning from the upstream long terminal repeat (LTR) to a sequence past the primer binding site, such as a sequence in the upstream region of the *gag* gene; the viral DNAs detected by such reactions are commonly referred to as late reverse transcription (LRT) products because they depend on the second template switch of reverse transcription for their formation [33,34]. Once reverse transcription is completed, IN hydrolyzes the extremities of the linear viral DNA adjacent to conserved cytosine-adenine dinucleotides located within the viral LTRs to generate reactive CA_OH_-3’ ends [35,36]; the resulting 3’-hydroxyl groups are subsequently used by IN to cut target DNA to effect viral DNA joining [37]. By convention, the integration-competent nucleoprotein complex formed by IN 3’ processing activity is referred to as the preintegration complex (PIC). HIV-1 PIC formation is believed to largely occur within the cytoplasm [38], as the virus traffics to the nuclear periphery along microtubules [32].

The majority of virus particles are noninfectious [39], and it is accordingly challenging to observe HIV-1 PIC nuclear transport directly with certainty. Nuclear transport is routinely inferred through comparison of steady-state levels of readily detectable markers of the bulk infection. Arguably, the most direct method is quantitative assessment of LRT DNA in cytoplasmic versus nuclear fractions. Importantly, this requires careful validation of the fidelity of cellular fractionation, using control protein or nucleic acid markers appropriate to represent the separate compartments [40,41]. While many fractionation procedures likely separate soluble cytoplasmic components adequately, protocols likely differ in their abilities to distinguish nucleoplasmic viral DNA from PICs associated with the NPC, or strongly associated with the cytoskeleton. Before successfully integrating into a host chromosome, a subset of PICs [42] are instead diverted to form non-productive circular DNA products through the action of host cell-mediated DNA repair pathways: 1-LTR circles can be produced by homologous recombination [43] or from aberrant reverse transcription [44,45], while 2-LTR circles are formed through non-homologous end joining of the viral DNA [46]. PCR primers that amplify products that span the LTR-LTR circle junction provide a convenient, albeit indirect measurement for assessing the competence of the virus to reach the nuclear interior. Due to the generation of reactive CA_OH_ ends by IN 3’ processing activity, a fraction of PICs aberrantly integrate their LTR ends back into an internal region of the viral DNA in a process referred to as autointegration [47,48,49,50]. Importantly, autointegrants formed by the insertion of one LTR end in the vicinity of the second viral DNA end can score as positive in assays quantitating 2-LTR circles, confounding the use of 2-LTR circle measures as readouts for nuclear viral DNA [50]. Specific aspects of PCR design, which take into account the DNA sequence at the LTR-LTR circle junction, can accordingly help to mitigate this complication [50]. Absolute and relative levels of integration can be measured through PCR reactions specific for integrated proviral DNA [34,51], while the distribution of integration sites along chromosomes are assessed by identifying sequences of viral-cellular DNA junctions within the infected cell population [52,53]. While continuous advancement of various microscopy-based approaches provides an important additional avenue to assess HIV-1 nuclear import and integration [31,54,55], high-throughput, live-cell approaches capable of kinetically witnessing individual nuclear import events are not yet available.

Various biochemical approaches have yielded insight into the viral proteins that remain as part of the viral nucleoprotein substrate for nuclear import. The RTC/PIC observed in the cytoplasm exceeds the diameter of the pore [31,32], so only a fraction of the viral and cellular proteins that associate with the PIC in the cytoplasm likely enter the nucleus [31]. The double-stranded reverse-transcribed viral DNA and a tetramer of IN protein form the heart of the PIC, as they comprise the intasome nucleoprotein complex that drives integration [56,57]. Keeping in mind that only one functional PIC is formed per infectious event, the identities of other PIC-associated viral proteins have been difficult to identify precisely. MA, RT, and Vpr were repeatedly found to be components of viral nucleoprotein complexes isolated from nuclear fractions [40,58,59,60,61]. A handful of studies found NC and PR as well [59,60,62], while CA was noticeably absent from many of these same studies [40,58,59,60,61,62]. In fact, CA was either observed to be absent [26,61], or found in only scant amounts [38] within viral complexes extracted from whole-cell or cytoplasmic extracts, prompting initial belief that the HIV-1 core uncoats completely prior to PIC formation. Subsequent microscopy studies have more readily observed CA in association with cytoplasmic nucleoprotein complexes [31,32], though the duration of this association remains largely unknown. One study found CA-staining ultrastructures associated with NPCs and the nuclear envelope [63], with the authors consequently suggesting that the majority of uncoating occurs at the nuclear periphery upon completion of reverse transcription. Although the extent of CA core integrity at the nuclear periphery is controversial, PIC-associated IN is at least partially exposed to the cytoplasm: cytoplasmically-localized fusion proteins containing green fluorescent protein (GFP) and the IN-binding domain of lens epithelium-derived growth factor (LEDGF)/p75 potently inhibited HIV-1 infection after reverse transcription [64]. While some studies have detected CA in the nuclear fraction following HIV-1 infection [65,66], it is not entirely clear how much of this signal represents intranuclear CA rather than CA protein associated with the nuclear envelope. It would therefore be instructive to determine how much of this signal co-fractionates with nuclear PICs.

## 3. Viral and Cellular Elements Implicated in HIV-1 PIC Nuclear Import

Many of the viral elements found in association with the PIC have been proposed to be important for HIV-1 nuclear import. Nuclear localization signals (NLSs) present in MA [67,68] and IN [60,69], as well as various non-canonical karyophilic signals in Vpr [61,70,71,72,73,74], have each been proposed to recruit cellular nuclear transport proteins. Basic-type NLSs within IN have been proposed to recruit KPN α adaptor proteins importin α1 (Rch1) [60] and importin α3 (KPNA4) [75], which would presumably require additional binding to KPN β1 for function (Figure 1). IN can also directly interact with KPN β proteins importin 7 [76,77] and transportin 3 (TNPO3, TRN-SR2, or importin 12) [54,78], though the relevance of these interactions have been brought into question [79,80,81]. IN and Vpr are additionally proposed to bind NUPs directly to facilitate nuclear import without the need for adaptor KPN carrier proteins, which include interactions between IN and NUP153 [82], and Vpr with Pom121 [70] or hCG1 [83].

The reverse transcribed genome is suggested to be an important determinant of HIV-1 PIC nuclear import, primarily through a triple stranded DNA flap element generated through the action of the central polypurine tract (cPPT) and central termination signal (CTS) [84,85]. While this element is not absolutely required for either nuclear import or infection [86,87,88], numerous groups have confirmed that the DNA flap exerts a positive effect during infection [89,90,91]. DNA plus-strand extension from the cPPT primer likely decreases the overall duration of reverse transcription within the cell [90,92], which may indirectly influence viral nuclear import during instances of limiting nucleotide concentrations, for example. Such a kinetic advantage to reverse transcription conferred by the cPPT is consistent with its ability to reduce the time-frame in which the viral single-stranded DNA is sensitive to the inhibitory activity of APOBEC3 cytosine deaminase restriction factors [93,94,95], and may similarly protect the RTC/PIC from other host defense proteins that could derail its trafficking [96].

While HIV-1 MA, IN, and Vpr NLSs can confer nuclear localization when fused to otherwise cytoplasmic proteins, some studies have refuted the importance of these signals in PIC nuclear import during infection [87,90,97,98,99]. Various features of HIV-1 biology can help to explain some of these discrepancies. Firstly, MA and IN in particular are not known to function as free proteins during the early phase of HIV-1 infection. Thus, studying MA or IN as recombinant proteins expressed in human cells may not uncover behaviors relevant to PIC biology. Secondly, many of the viral constituents of the PIC are multifunctional proteins, whereby mutations may result in multiple coincident defects to infection, obfuscating the targeted assessment of contributions to a particular phenotype. Lastly, there is little evidence to support that HIV-1 enters the nucleus during mitosis when its passage through the NPC is blocked [100]. Thus, the historical perspective that HIV-1 nuclear import mutants would specifically be blocked for infection of non-dividing target cell types would since appear to be largely misguided.

## 4. CA Functionally Determines Requirements for Nuclear Trafficking

**Figure 3:**
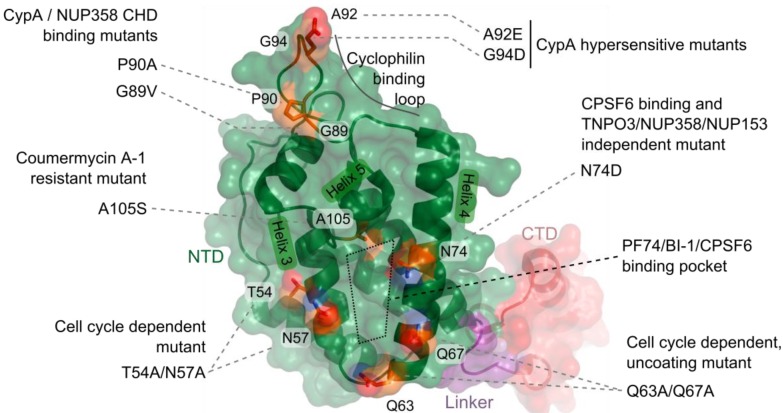
Schematic of HIV-1 capsid (CA) and mutations described in the text. A single HIV-1 CA monomer (protein database code 3j34) is represented by a cartoon of the peptide backbone, as well as a semi-transparent surface representation: N-terminal domain (NTD), green; flexible linker, purple; C-terminal domain (CTD), red. A subset of CA residue side-chains that exhibit phenotypic differences in preintegrative steps of HIV-1 infection are shown as sticks and colored as follows: carbon, orange; nitrogen, blue; oxygen, red.

The field has more recently moved to view CA as the major viral protein that mediates HIV-1 nuclear import. Masahiro Yamashita and Michael Emerman demonstrated that infection by an HIV-1 chimeric virus that carried the MLV CA protein was cell cycle dependent [101], mimicking the property of parental MLV. The defect to infection upon cell cycle arrest was at the step of nuclear import, as a decrease in the formation of 2-LTR circle DNAs relative to wild-type (WT) HIV-1 was observed [101]. They subsequently determined that certain point mutations in HIV-1 CA, including T54A/N57A and Q63A/Q67A (Figure 3), also imparted cell cycle dependence to HIV-1 [41]. Notably, the infection defect exhibited by the T54A/N57A mutant virus upon cell cycle arrest occurred after nuclear entry but before integration [41]. While this mutant was sensitive to cell cycle arrest in all cell lines tested, the growth arrest phenotypes of A92E and G94D CA mutant viruses, which are hypersensitive to the levels of CA-interacting host protein cyclophilin A (CypA) in certain cell lines (HeLa and H9 cells), were restricted to these same cells [102,103]. Cell cycle arrest also inhibited these viruses after nuclear import, as both LRT and 2-LTR circle levels remained unchanged. While the infection defects experienced by these CA mutant viruses upon cell cycle arrest occur following HIV-1 nuclear entry, the Q63A/Q67A mutant virus appeared to be defective for nuclear import, as it exhibited decreased levels of 2-LTR circle formation as compared to WT virus in dividing cells [104]. Q63A/Q67A CA cores recovered from whole virions following detergent treatment were less stable than WT cores *in vitro* [105], but the mutant viral RTCs and PICs retained a greater complement of CA protein than did the WT virus during infection [27,41,104]. This apparent delay in Q63A/Q67A core uncoating appears related to the nuclear import and integration defects experienced by this mutant virus.

In addition to CypA, other CA-interacting host factors have been observed to affect HIV-1 nuclear import and integration. The rhesus Trim5α restriction factor normally restricts HIV-1 infection by targeting the viral core for disassembly and degradation prior to the completion of reverse transcription [106,107]. Inhibition of cellular proteasome activity with the small molecule MG132 rescued reverse transcription while having no effect on the ultimate level of integration [108]. The MG132-rescued RTCs seemingly remain intact [109] and mature into integration-competent PICs [110]. These complexes also escape entrapment by proteasome-associated cytoplasmic bodies [111] yet accumulate fewer 2-LTR circles, consistent with a trafficking defect coincident with or shortly prior to nuclear import [108,110]. Various artificially engineered Trim-CypA fusion constructs have also been shown to cause defects to infection after reverse transcription, though these appear to occur after nuclear entry [112]. Similar phenotypes have been observed with variants of the cleavage and polyadenylation specific factor 6 (CPSF6) mRNA processing protein. While expression of the C-terminal truncation variant CPSF6_375_ inhibited HIV-1 reverse transcription [113], the marginally shorter C-terminal truncation mutant CPSF6_358_, which harbored amino acid residues oftentimes removed as exon 6, inhibited 2-LTR circle formation while leaving reverse transcription unaffected [114,115]. Recently, the interferon induced antiviral protein Mx2 was found to block HIV-1 infection after reverse transcription in a CA-dependent manner [116,117].

Similar phenotypes have recently been observed with small molecules that target HIV-1 CA. Pfizer compound PF-3450074 (PF74), which was discovered in a high-throughput screen for inhibitors of HIV-1 replication [118], was subsequently shown to bind a hydrophobic pocket formed between HIV-1 CA NTD alpha helices 3, 4, and 5 [118] (Figure 3). PF74 destabilized virion-purified CA cores *in vitro*, and inhibited the completion of reverse transcription during infection [119]. Boehringer Ingelheim pyrrolopyrazolone compounds BI-1 and BI-2 were more recently determined to engage the same pocket within the CA NTD [120]. Interestingly, treatment with these compounds did not affect HIV-1 reverse transcription, but instead resulted in decreased accumulation of 2-LTR circles. The phenotypic difference may relate to potentially contrasting effects on CA uncoating, as BI-1 delayed the disassembly of higher-order HIV-1 CA-NC structures *in vitro* [120]. Interestingly, the binding sites of these compounds on CA overlap that of CPSF6 [120,121] and NUP153 [122]. Coumermycin A-1, a separate small molecule, was found to inhibit HIV-1 integration [123]. While the binding site of this compound is not known, passage of HIV-1 in its presence yielded the outgrowth of virus encoding the A105S mutation in the CA NTD. As Ala105 is proximal to the aforementioned PF74/BI-1/CPSF6 binding site (Figure 3), it seems likely that the inhibitory mechanism of coumermycin A-1 is related to these factors.

Perturbation of CA can also affect the post-reverse transcription steps of other retroviruses. Though MLV does not enter the nucleus via the NPC, it must also in large part dissolve its CA shell to effect IN-mediated integration. MLV CA is readily found in RTCs purified from infected cells, and remains stably associated with the PIC until it enters the nucleus [124]. MLV p12, a *gag*-encoded protein not found in HIV-1, is crucial for nuclear targeting as it tethers PICs to mitotic chromosomes [125,126,127]. While MLV CA dissociates from the PIC during mitosis, p12 mutants PM14 and S61A/S65A, which are defective for mitotic chromosomal tethering, each maintain CA in association with the PIC during mitosis [128]. Certain murine cells take advantage of RTC/PIC-associated CA to interrupt the MLV infection mechanism: the protein expressed from Friend virus susceptibility-1 (*Fv1*), which is a *gag*-related gene from a murine endogenous retroelement [129], is able to target MLV cores to inhibit infection [130,131,132]. Restricted MLV still reverse transcribes and forms PICs capable of integrating *in vitro* [133], yet does not form circular DNA byproducts [134,135]. The product of the *Fv1* gene can also inhibit HIV-1 infection when targeted to HIV-1 CA upon fusion to CypA, resulting in a quantifiable decrease in the number of integrated proviruses while leaving the accumulation of 2-LTR circles unchanged [136].

Together, these results show that the retroviral core shell is unlikely to passively fall apart upon viral entry, but instead functions at a critical juncture bridging reverse transcription and nuclear trafficking. Although premature CA disassembly and proteasomal targeting may exert their effects as early as reverse transcription, other perturbations to CA uncoating and CA-determined trafficking defects prevent PIC nuclear import, or even manifest as defects within the nucleus. MLV has specifically evolved to access chromosomes during mitosis, and accordingly, the combined functions exerted by MLV CA and p12 likely specifically link MLV uncoating and nuclear entry with mitosis. In the aforementioned chimeric HIV-1 encoding MLV *gag*, the PIC is likely forced into an MLV-type mechanism of nuclear entry. Contrastingly, the cell cycle dependence of HIV-1 CA missense mutations may be due to reasons stemming from various potential losses in function: for example, perturbed core engagement with host proteins may result in a virus blocked at one of many preintegrative steps of infection, and may require cellular rearrangements that occur during cell division to relieve this block. While the previously described phenotypes reveal effects the retroviral core may exert on steps following reverse transcription, recent findings that CA protein physically associates with nuclear transport factors hints that CA takes a direct role in promoting the nuclear steps of HIV-1 infection.

## 5. CA-Associated Host Proteins that Promote HIV-1 Nuclear Import

A series of genome-wide RNA interference screens [17,18,19,20] identified numerous nuclear transport factors as potential HIV-1 cofactors: TNPO3, NUP358/RANBP2, NUP155, NUP153, and NUP98 were each identified in two independent screens, while a number of additional NUPs (NUP50, NUP62, NUP85, NUP107, NUP133, NUP160, NUP210, NUP214, ELYS, and TPR) and soluble transporters (KPN β1, XPO1, and NXF1) each hit once [17,18,19,20,137] (Figure 1). The HIV-1 requirements for TNPO3, NUP358, and NUP153 mapped to the nuclear steps of infection, either preceding or concomitant with integration [18]. Knockdown of NUP358 [66,138,139] or NUP153 [66,140] yielded fewer 2-LTR circles, though additional defects to integration could not be ruled out. While defects to integration were readily confirmed between the studies, the impact of TNPO3 knockdown on HIV-1 nuclear import is less certain; there has been little consensus between studies on whether 2-LTR circle levels drop [54,138,141] or remain unchanged [65,142,143] during infection. It was recently proposed that an increase in viral autointegration upon TNPO3 knockdown masks the 2-LTR defect detected with conventional PCR primers [50].

Depletion of nucleocytoplasmic transport proteins alters the integrity of NPC structure and function to varying levels [144,145,146], and likely triggers a range of systems-level effects altering the subcellular localization of countless host proteins. Furthermore, many NUPs and KPNs play major roles during mitosis [147,148,149]. Accordingly, there is valid concern that some of the infection phenotypes observed in knockdown studies are due to indirect effects caused by global perturbation of cellular activity. At least for TNPO3, NUP358, and NUP153, such concerns are somewhat assuaged by subsequent findings which demonstrated that the knockdowns affected HIV-1 infection in relatively specific, CA-dependent manners. Firstly, only a subset of retroviruses appears to utilize these proteins for infection. HIV-1 is dependent, consistent with its ability to usurp the cellular nuclear import machinery, while MLV is unaffected by depletion of any of these proteins [18,80,139,140,150]. This difference is however not simply due to the contrasting nuclear targeting mechanisms between retroviral genera, as the lentivirus feline immunodeficiency virus (FIV) was similarly resistant to the effects of NUP153 or TNPO3 depletion [80,114,140,141,150], with equine infectious anemia virus (EIAV) yielding an intermediate phenotype [80,140,141,150]. Secondly, the contrasting dependencies between HIV-1 and MLV afforded tests of the previously mentioned HIV-1/MLV chimera viruses, which revealed CA to be the main determinant for NUP153 and TNPO3 dependence [80,140]. Thirdly, specificity for factor requirements could be traced to individual amino acid residues within CA; notably, the N74D mutation conferred insensitivity to knockdown of TNPO3, NUP358, or NUP153 [114,139,140]. Expanded CA mutant virus panels found additional mutations that conferred resistance to NUP153 or TNPO3 depletion [140,142]. Lastly, co-depletion of certain other host factors, such as CypA, could fully counteract the observed infection defect [50,139,140,151].

### 5.1. Potential Roles of TNPO3 in HIV-1 Infection

Shortly following the identification of TNPO3 by Brass and colleagues [17], TNPO3 was independently identified as an IN interacting protein using a yeast two-hybrid screen [54]. While the initial report showed TNPO3 preferentially bound HIV-1 over MLV IN [54], subsequent reports found TNPO3 to bind both proteins comparably [80,150]. In fact, TNPO3 efficiently bound a variety of purified retroviral IN proteins [80], including those derived from TNPO3-independent viruses, such as MLV and FIV. HIV-1 IN interacts with TNPO3 with relatively high affinity, with measured dissociation constants ranging from ~17 nM [152] to ~260 nM [80]. This interaction could be competed by the Ran GTPase mutant Q69L in complex with GTP, consistent with IN being an import cargo for TNPO3 [153]. A series of basic residues within the IN CTD, including Arg262 and Lys264, appear particularly important in binding to TNPO3 [78,152]. Unfortunately, viruses containing IN proteins harboring TNPO3 binding mutations exhibited pleiotropic defects during infection, which included greatly reduced levels of reverse transcription, precluding the specific measurement of associated nuclear import defects [152]. Notably, a chimeric HIV-1 virus harboring MLV IN in place of HIV-1 IN was as sensitive to TNPO3 depletion as WT HIV-1 [80,150]. While IN may engage TNPO3 in the context of HIV-1 infection, the potential role for this interaction in PIC nuclear localization appears auxiliary to that played by CA.

Various reports have investigated whether CA might bind to TNPO3 directly. A recombinant fusion protein consisting of glutathione *S*-transferase and TNPO3 pulled-down CA and cellular tRNA from purified virions [65]. The extent of CA recovery was enhanced in the presence of Ran(Q69L)-GTP, implying a directionality of binding favoring nuclear export, though the quantitative difference in this result was only approximately two-fold [65]. TNPO3 preferentially bound WT over TNPO3-independent N74D mutant CA-NC tubes *in vitro* [143], though this difference was less than two-fold, and a subsequent study failed to confirm this difference [78]. TNPO3 binding has been suggested to alter CA core uncoating: purified TNPO3 accelerated the extent of *in vitro* uncoating of purified viral cores, while Ran(Q69L)-GTP counteracted this effect [154]. It remains unclear whether the relatively weak *in vitro* interaction between HIV-1 CA and TNPO3 occurs within cells, and if it does, whether it is relevant for infection.

There is some evidence to suggest that the primary function of TNPO3 during HIV-1 infection is control of CPSF6 localization within the cell. CPSF6 is a member of a large family of eukaryotic pre-mRNA splicing factors collectively referred to as SR proteins. These proteins typically contain discrete domains rich in arginine-serine dipeptide repeats (RS domains). RS domains have been shown to serve as nuclear localization signals for other SR proteins involved in mRNA splicing, such as ASF/SF2 and SC35 [155], by interacting with TNPO3. While an interaction between CPSF6 and TNPO3 has yet to be formally shown, it appears as though TNPO3 does bind CPSF6 and control its localization: CPSF6 is normally a nuclear protein, and TNPO3 knockdown has been observed to increase the redistribution of CPSF6 to the cytoplasm [50]. While exogenously introduced full-length CPSF6 is predominantly nuclear and does not adversely affect HIV-1 infection [113,114], mislocalizing it to the cytoplasm by appending a nuclear export sequence inhibits infection [151]. Conversely, attaching a functional NLS onto the normally cytoplasmic CPSF6_358_ counteracted its ability to restrict HIV-1 infection [50,121]. Concomitant CPSF6 knockdown moreover counteracted the inhibitory effects of TNPO3 depletion [50,151], and a similar set of CA mutations conferred insensitivity to TNPO3 depletion and CPSF6_358_ restriction [50]. Still, some inconsistencies remain: for example, EIAV does not appear to bind CPSF6 [115,151], yet it is reproducibly inhibited by TNPO3 depletion [80,141,150]. While TNPO3 may serve more than one role during HIV-1 infection, altered CPSF6 localization seems to account for a major part of the infectivity defect induced by TNPO3 knockdown.

### 5.2. Viral Interactions with NUP153 and NUP358

The roles of NUP153 and NUP358 during HIV-1 nuclear import have also been recently investigated. NUP153 is a large 1475 amino acid NUP found on the nuclear side of the NPC. While its NTD associates with the nuclear basket [156,157], its highly flexible CTD can reach into the central channel [158], extending across to the cytoplasmic side of the NPC in a transport-dependent manner [159,160,161]. Like TNPO3, NUP153 was reported to bind HIV-1 IN [82]. This interaction was mapped to the C-terminal FG-rich domain of NUP153. Though interaction could be observed between purified NUP153 CTD and HIV-1 IN, it was not observed with FIV IN [82]. Additionally, this interaction was inhibited by the addition of cytosolic extract, presumably due to competition with KPN binding to the NUP153 CTD, as addition of a non-hydrolyzable GTP analog counteracted this effect [82]. This result may explain why co-immunoprecipitation was not observed between cell expressed GFP-tagged NUP153 and HIV-1 IN [162]. An interaction was also observed between GFP-NUP153 extracted from animal cell lysate and CA-NC tubes *in vitro* [162]. We recently confirmed this finding, and have additionally detected a direct interaction between the NUP153 CTD and HIV-1 CA NTD [122]. This interaction moreover mapped to the FG motifs present within the NUP153 CTD [122]. Notably, NUP153 bound N74D CA as well as WT CA [122,162].

NUP358 is an even larger, 3224 amino acid NUP that forms the cytoplasmic filaments emanating from the NPC [163,164,165] (Figure 1). In contrast to TNPO3 and NUP153, the only retroviral protein NUP358 has been published to bind is HIV-1 CA [139]. This interaction was found to occur through the CypA homologous domain (CHD) that resides at the C-terminus of NUP358. The NUP358 CHD is similar to CypA in both overall primary sequence and tertiary structure, though there are a few differences in active site residues [166]. NUP358 was initially found to bind the CA NTD with slightly weaker affinity than CypA (16 μM, vs. 7 μM for CypA) [139], though subsequent reports indicate a much weaker interaction (~100–200 μM) [166,167]. The CA proteins from SIVmac and HIV-1 CypA loop mutants G89V and P90A do not bind CypA [168], and these proteins also failed to bind NUP358 CHD [139]. These viruses were accordingly insensitive to NUP358 knockdown [139]. Consistent with its localization to the cytoplasmic edge of the NPC, NUP358 appears to be critical for the docking of the HIV-1 PIC to the NPC during infection [66]. NUP358 CHD appears to possess isomerase activity for various substrates [166,169]. Although it is capable of isomerizing HIV-1 CA [167], it is not yet clear whether isomerization is necessary for HIV-1 infection. Additionally, other regions of NUP358 aside from the CHD may serve important functions during infection [170].

Interestingly, NUP153 and NUP358 exhibit certain similarities. While found on opposite sides of the NPC, each protein localizes to the periphery of the NPC, and may represent initial sites of NPC attachment/detachment during passage through the nuclear pore. Accordingly, each protein contains FG repeats, and makes contacts with nuclear transport receptors. NUP153 and NUP358 each encode tandem repeats of homologous zinc-fingers, which are involved in Ran binding [171,172] and COPI recruitment during nuclear envelope breakdown [173]. It remains to be seen whether any of these similarities play into the importance of these proteins during HIV-1 infection.

### 5.3. Interdependence of CA-determined Host Factors during Infection

A number of phenotypic similarities suggest the roles of TNPO3, NUP153, NUP358, CPSF6, and CypA during HIV-1 infection are interrelated. Starkly, the N74D CA mutant virus is insensitive to knockdown of TNPO3, NUP153, and NUP358, despite possessing a CA protein capable of binding all three proteins with similar affinities to WT CA. As the N74D mutation clearly counteracts CPSF6 binding [114], it seems plausible that CPSF6 engagement licenses HIV-1 to employ NUP358 and NUP153 during infection. CPSF6 is currently believed to be exclusively nuclear at steady state, suggesting that it may not exert its effects on HIV-1 until the virus engages the NPC. Curiously, siRNA depletion of CPSF6 does not affect HIV-1 infection [114].

CypA also appears to alter nuclear transport factor dependence during HIV-1 infection. Abrogation of CA binding with CypA, either through CypA depletion or competition with the small molecule cyclosporine, rescued viruses inhibited by NUP153 or NUP358 knockdown [139,140]. While cyclosporine treatment can partially rescue WT HIV-1 infection in TNPO3 depleted cells [139,154], the lack of complete rescue may reflect its multiple potential roles in promoting HIV-1 infection. CypA binding to HIV-1 CA can alter its disassembly [154,174], suggesting that its effect on NUP153, NUP358, and TNPO3 may be indirect through modulating the rate and extent of CA core uncoating. Indeed prevention of CypA binding also modulates the antiviral effects of the rhesus Trim5α [175] and Mx2 [116,117] restriction factors. Though CypA serves as a major regulator of the pre-integrative steps of HIV-1 infection, the precise mechanistic details by which CypA exerts its effects are not well understood.

## 6. Effects of Nuclear Transport Proteins on Integration Site Selection

While HIV-1 appears to predominantly utilize NUP153, NUP358, and TNPO3 to affect its import into the nucleus, these factors can also affect post-nuclear trafficking as evidenced by differences in HIV-1 integration site distributions upon factor knockdown. Numerous different forces can influence integration site distribution. IN favors certain nucleotide patterns at the site of integration [176,177]. Integration also favors the distorted major grooves that occur when DNA is wrapped around the nucleosome core [178,179], as well as certain epigenetic modifications [179]. On the genomic level, HIV-1 preferentially targets the bodies of active genes within gene dense regions of chromosomes [52]. Whereas LEDGF/p75 in large part dictates the preference for active gene bodies [180,181,182], the targeting of gene dense regions of chromosomes is apparently linked to nuclear import: depletion of TNPO3, NUP358, and to a lesser extent, NUP153, significantly reduced the extent of integration in gene dense regions of chromatin [139,162,183,184]. This pattern was moreover consistent with the involvement of CA, as the HIV-1 chimeric virus encoding MLV CA, as well as CA missense mutants N57A and N74D, showed a similar shift in integration site distribution [139,183,184]. Notably, CypA binding was also found to affect integration site selection, as disruption of CypA binding to CA by cyclosporine treatment resulted in an increase in the number of integration events in chromosomal regions enriched in transcriptional units [139].

It is possible that these proteins are directly involved in guiding the PIC to distinct regions of chromatin; NUP153 has been shown to associate with large regions of active chromatin in drosophila [185], and TNPO3 may engage the HIV-1 intasome to effect integration [78]. On the other hand, NUP358 appears more important for integration targeting than NUP153, yet this protein does not appear to be found within the nucleus during interphase [186]. Furthermore, depletion of a number of other nuclear host proteins including IK, ANAPC2, WDHD1, SNW1, and PRPF38A similarly redirected integration site targeting [183]. It remains formally possible that the roles of some host factors in dictating integration to gene dense regions may be indirect, instilled through alteration of global chromosomal environment as compared to specific effects on HIV-1 PIC trafficking. Still, ablation of gene-dense region targeting by CA mutations such as N74D highlights a specific role for CA in post-nuclear PIC trafficking. The mechanism of nuclear import may be linked with integration site targeting by affecting the chromosomal environments first encountered by the PIC upon nuclear entry.

## 7. Model of CA and Nuclear Transport Factors during HIV-1 Nuclear Entry

**Figure 4:**
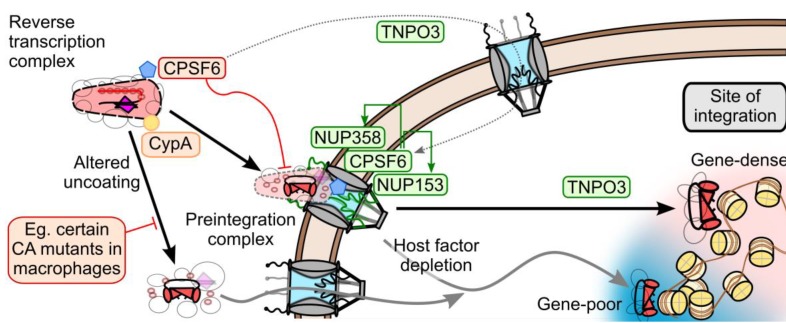
Model of the potential roles of the CA-dependent nuclear transport factors during HIV-1 infection. NUP358, NUP153, and CPSF6 at the nuclear pore most likely act on PIC-associated CA to aid HIV-1 infection. TNPO3 is required to localize CPSF6 to the nucleus; premature cytoplasmic CPSF6 binding to CA prevents nuclear import. TNPO3 may affect integration by interacting with IN within the nucleus. CypA modulates CA uncoating, altering dependencies on NUP358, NUP153, and TNPO3. Perturbation of this pathway by CA mutation or TNPO3, NUP153, or NUP358 knockdown results in altered integration site selection away from gene-dense regions of chromatin.

We propose the following working model to coalesce recently reported results from the rapidly evolving field of HIV-1 PIC nuclear transport (Figure 4). While the initial steps of uncoating likely occur shortly after entry [27], the final events of uncoating may occur at the NPC [63]. The partially uncoated PIC most likely docks at the NPC, by engaging NUP358 with its remaining CA proteins [66,139]. Once docked, CPSF6 and NUP153 then engage the PIC. The combined actions of these proteins are necessary for PIC nuclear import. TNPO3 expression is required for proper nuclear localization of CPSF6; CPSF6 binding to CA cores too early during infection misregulates the upstream steps of uncoating and NPC engagement, blocking infection at the step of nuclear import. TNPO3 may also have an additional intra-nuclear role permitting proper nuclear trafficking and integration, perhaps related to its interaction with IN. These concerted steps of uncoating and nuclear import appear to influence the downstream steps of nuclear trafficking and integration, as depletion of TNPO3, NUP153, and NUP358 reduce targeting of the PIC to gene-dense regions of chromatin. It will be instructive to ascertain if CPSF6 depletion influences HIV-1 integration site distribution.

The precise mechanistic requirements for NUP358, NUP153, and CPSF6 for nuclear import remain unclear: these proteins may be critical for a prerequisite uncoating step prior to nuclear import, or they may be directly involved in the act of PIC nuclear translocation. As these three proteins have each been published to bind CA, the latter model presupposes that CA would need to be concomitantly imported into the nucleus with the PIC. This point remains highly controversial; CA has historically been noticeably absent from the nucleus, with only a couple recent reports observing potential PIC-associated CA signals within the nuclear fraction [65,66]. Additionally, there is currently no evidence describing a mechanism by which disassembled CA may remain associated with the PIC. Notably, TNPO3, NUP358, and NUP153 are not absolutely required for HIV-1 infection of transformed cell lines: though WT virus is highly dependent on these factors, CA mutant viruses such as N74D can bypass CPSF6 binding and infect cells depleted for NUP358, NUP153, or TNPO3 without a concomitant loss of infectivity. While the N74D CA mutant virus was previously proposed to bypass these requirements by relying on an alternative set of NUPs (including NUP155 and NUP98) [114], it is not clear whether these proteins indeed fulfill critical roles for N74D mutant virus infection [162].

Alternatively, if the main function of these nuclear transport factors is to uncoat the PIC as a prelude for nuclear import, then alterations to viral uncoating may obviate the need for this mechanism during infection. While a mechanism for active PIC nuclear transport would be required in all cell types, optimal CA uncoating may be particularly important in particular cells, such as macrophages, where premature uncoating may render the PIC susceptible to intracellular antiviral factors. Indeed, the N74D CA mutant virus exhibits a significant infectivity defect in monocyte-derived macrophages [139,187], where its reverse transcription is defective [187]. Because Asn74 is highly conserved among primate lentiviruses, HIV-1 may very well rely on these nuclear transport factors *in vivo* [114]. The HIV-1 requirement for CypA may be similarly nuanced: while it exerts differential effects in various cell types, its importance as a fine-tuned regulator of CA uncoating is likely most important in cell types where premature uncoating may be most detrimental [139,188].

Curiously, although HIV-1 appears to rely upon NUP358, NUP153, TNPO3, and CPSF6 during infection, other lentiviruses only appear to share certain aspects of this mechanism. SIVmac does not bind NUP358, and accordingly does not rely on this protein for infection. Furthermore, EIAV utilizes NUP153 and TNPO3, though it does so in the apparent absence of CPSF6 binding. FIV likely utilizes an entirely different mechanism, as it does not seem to require any of these factors. Similar to CypA [189], various lentiviruses may have evolved to differentially rely upon these nuclear transport factors over time. Although recent years have witnessed significant advances on the role of CA and particular nuclear transport proteins in HIV-1 PIC nuclear import, there is clearly much left to learn about how HIV-1 and some of the other lentiviruses circumvent the nuclear envelope to reach their chromosomal targets of integration.

## 8. Conclusions

Despite intense study, the details of HIV-1 and other lentiviral nuclear import mechanisms remains incompletely understood. This is perhaps at least somewhat reflective of the complexity and potential redundancy of nuclear transport mechanisms through the NPC, as well as difficulties in precisely measuring or developing accurate *in vitro* approximations for a trafficking process that occurs at the heart of the lentiviral replication cycle. The convergence of recent findings functionally relating the requirements of the retroviral CA protein with cellular nuclear transport factors have provided new insight into the series of interrelated steps that begin with HIV-1 CA uncoating and end with proviral integration. Despite its intricacies, continued study supported by advancements in genetic, biochemical, and microscopy approaches are predicted to eventually unravel the detailed molecular mechanisms that underlie retroviral PIC nuclear import.
